# Beyond nutritional risk scores: domain-guided perioperative nutrition in patients with pancreatic ductal adenocarcinoma undergoing pancreaticoduodenectomy

**DOI:** 10.3389/fnut.2026.1955662

**Published:** 2026-10-21

**Authors:** Jingyi Xu, Lei Yang, Yuehua Liang, Xialin Xie, Wenqiang Wang, Lu Gao, Bo Jia, Jingjing Li, Jun Yan

**Affiliations:** 1 Department of Liver Surgery, Beijing Tsinghua Changgung Hospital, School of Clinical Medicine, Tsinghua Medicine, Tsinghua University, Beijing, China; 2 Department of Basic Medicine, Beijing Health Vocational College, Beijing, China

**Keywords:** cancer cachexia, pancreatic ductal adenocarcinoma, pancreatic exocrine insufficiency, pancreaticoduodenectomy, perioperative nutrition, sarcopenia

## Abstract

Patients with pancreatic ductal adenocarcinoma (PDAC) proceeding to pancreaticoduodenectomy rarely have a single nutritional problem. Poor intake may coexist with pancreatic exocrine insufficiency, loss of muscle function, systemic inflammation, and unstable glucose control. Screening tools identify vulnerability, but they do not show which of these processes is most important at a particular point in treatment. This Perspective sets out a domain-guided approach in which screening is followed by an explicit treatment hypothesis. Four overlapping domains are considered: muscle loss and functional decline; pancreatic exocrine insufficiency and impaired digestion; inflammatory and catabolic burden; and glycemic instability with pancreatic endocrine insufficiency. The domains are not new diagnoses and do not replace the Nutrition Care Process. They are a practical way to connect a clinically important finding with a defined action, a responsible clinician, a response measure, an escalation rule, and a reassessment date. Intake and symptom burden are treated as cross-cutting variables because they influence every domain. The framework is intended for use during neoadjuvant treatment, the preoperative interval, hospitalization, and early recovery. Its value will depend on whether it improves function, symptom control, protein-energy adequacy, glycemic stability, postoperative recovery, and delivery of planned oncologic treatment.

## 1 Introduction

Pancreaticoduodenectomy (PD) is undertaken in patients whose nutritional reserve is often already compromised. Reduced oral intake, pancreatic exocrine insufficiency (PEI), diabetes or prediabetes, biliary obstruction, chemotherapy-related symptoms, systemic inflammation, and physical deconditioning commonly coexist before surgery. Current surgical and oncology nutrition guidance rightly emphasizes early screening, repeated assessment, individualized nutrition therapy, early oral or enteral feeding when feasible, glucose management, and mobilization (1–9). In pancreatic ductal adenocarcinoma (PDAC), however, these measures must be integrated with neoadjuvant treatment, recovery from a high-risk operation, and the need to start adjuvant therapy without avoidable delay (4–7).

The unresolved problem is what follows a positive screen. A high Nutritional Risk Screening 2002 score, a GLIM diagnosis, a low prognostic nutritional index, or reduced CT-derived skeletal muscle area identifies vulnerability, but it does not explain why the patient is deteriorating. The same score may reflect insufficient intake in one patient, untreated maldigestion in another, or inflammation-driven wasting in a third. These mechanisms are clinically distinct. Prescribing more calories without establishing which process is dominant risks treating the measurement rather than the patient.

This distinction is especially important in PDAC, where several plausible causes of weight and functional loss often operate at the same time. A patient may tolerate oral supplements yet continue to lose weight because the pancreatic enzyme dose is inadequate. Another may maintain body weight during neoadjuvant therapy while chair-rise performance declines and activity falls. A third may have adequate prescribed intake but remain catabolic because of recurrent cholangitis or progressive disease. Such cases are not exceptions; they illustrate why a prognostic label is an incomplete basis for treatment.

Although PD is performed for several periampullary and benign conditions, PDAC warrants separate consideration. Neoadjuvant chemotherapy, tumor-related cachexia, biliary intervention, and the narrow interval before adjuvant treatment create a sequence of nutritional hazards that is not captured by the operation alone. Evidence from mixed surgical cohorts remains relevant, but it should inform rather than define a PDAC-specific pathway. The framework is therefore anchored to treatment stage as well as to surgical anatomy.

We therefore argue for a shift from risk classification to an explicit treatment hypothesis. The four overlapping domains—muscle loss and functional decline; PEI and impaired digestion; inflammatory and catabolic burden; and glycemic instability with pancreatic endocrine insufficiency—are not new diagnoses or novel measurements. Their practical value is to convert familiar findings into parallel, treatment-oriented hypotheses, each linked to an intervention, a designated clinician or team, a response measure, and a reassessment point. This structure is intended to prevent a high-risk score or a single abnormal test from becoming the endpoint of assessment while preserving attention to interactions among domains.

## 2 What domain-guided care adds to existing frameworks

The Nutrition Care Process (NCP) already provides a disciplined sequence of assessment, diagnosis, intervention, and monitoring (10). The present framework sits within that process. Its PDAC-specific contribution is a structured approach to clinical prioritization when several abnormalities coexist: the team identifies the domain most likely to change the next clinical decision while retaining a core concurrent assessment of the other domains. Prioritization therefore guides sequencing and responsibility without narrowing the clinical assessment.

A domain is best understood as a cluster of findings that changes management. The four domains are intentionally not identical in type. Muscle loss is a phenotype with functional consequences; PEI is a digestive mechanism; inflammatory and catabolic burden describes a pathophysiologic setting; and glycemic instability is a metabolic management problem. That asymmetry is acceptable because the aim is treatment selection, not etiologic taxonomy. Intake and symptom burden are treated as cross-cutting variables rather than a fifth domain, since they can initiate, amplify, or obscure each of the other four. Figure 1 summarizes this staged approach.

**Figure 1:**
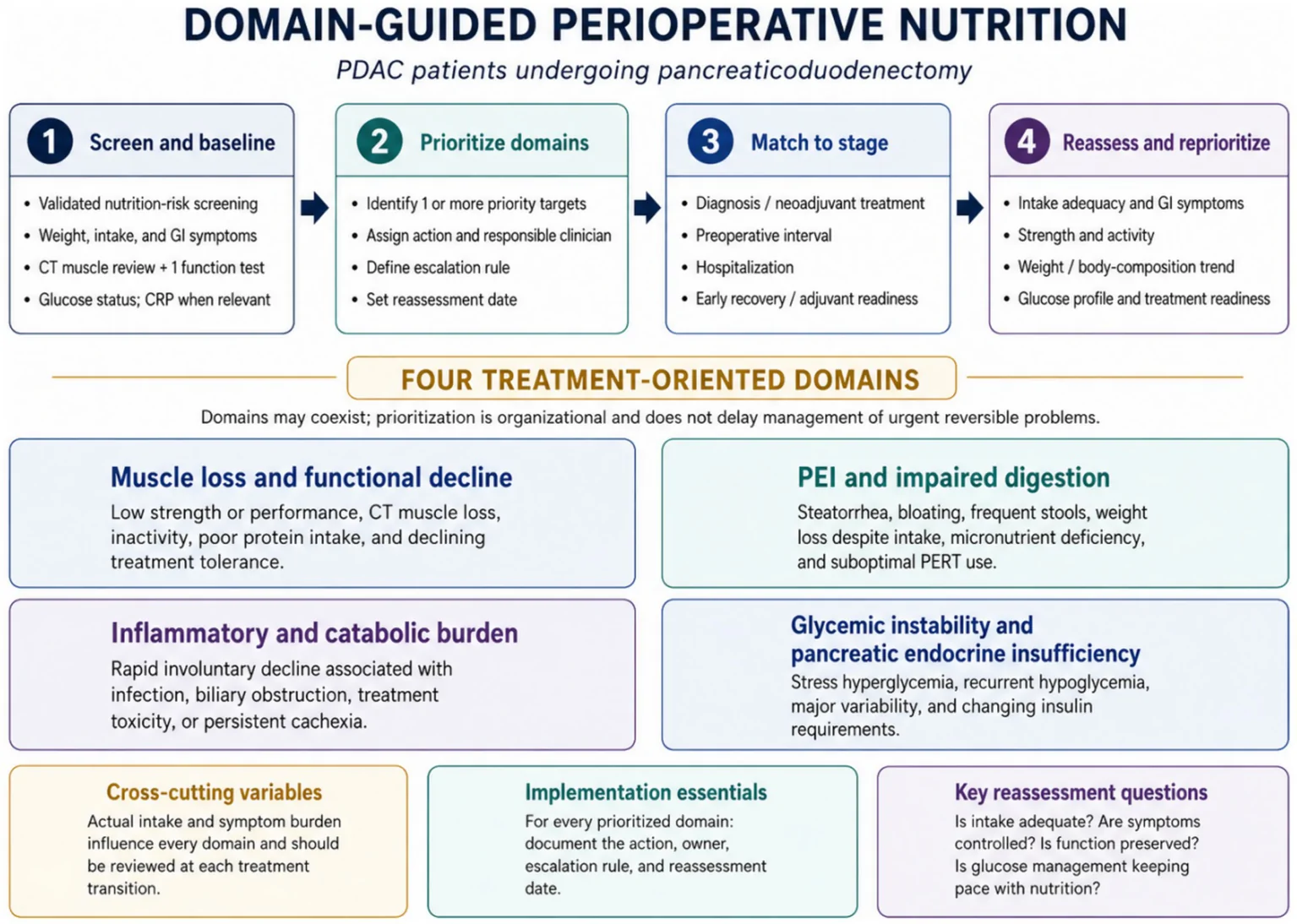
Domain-guided pathway for perioperative nutrition in patients with pancreatic ductal adenocarcinoma undergoing pancreaticoduodenectomy. Routine screening and baseline assessment identify vulnerability; focused assessment identifies one or more priority treatment-oriented domains; interventions are matched to the current treatment stage; and response is reassessed to allow reprioritization. Prioritization is organizational: all four domains remain under core surveillance, and urgent reversible problems are managed in parallel. CRP, C-reactive protein; CT, computed tomography; GI, gastrointestinal; PEI, pancreatic exocrine insufficiency; PERT, pancreatic enzyme replacement therapy.

We propose three pragmatic considerations for prioritization: whether an abnormality requires a change in management now; whether a modifiable component can be addressed before the next oncologic milestone; and whether delay could compromise safety, nutritional adequacy, function, or treatment delivery. Urgent reversible problems—sepsis or cholangitis, biliary obstruction, severe hypo- or hyperglycemia, and major feeding failure—take precedence over elective priorities. Otherwise, one or more domains may be designated primary when they have the clearest near-term treatment consequence, with other active problems managed in parallel.

Prioritization is time-dependent rather than exclusive. At each reassessment, a core cross-domain review should still include actual intake and symptoms, weight and functional trajectory, digestion and PERT use, inflammatory context, and glucose pattern. For each active priority, the record should state the intended action, designated clinician or team, response measure, escalation threshold, and reassessment date. Failure to improve, or deterioration in another domain, should prompt reprioritization rather than automatic intensification of the original intervention.

The same finding may carry different weight across the cancer pathway. Low muscle mass at diagnosis may justify early protein support and exercise counseling; continued decline during neoadjuvant therapy has greater urgency because it can threaten operative or adjuvant readiness. A CRP rise during documented cholangitis should be interpreted differently from persistent inflammation and functional loss after source control. Clinical stage before surgery, pathological stage after resection, treatment intent, and extent of pancreatic resection should therefore be recorded as context for prioritization. Table 1 summarizes these decision rules.

**Table 1:** Focused clinical decision matrix for four treatment-oriented nutrition domains in patients with pancreatic ductal adenocarcinoma undergoing pancreaticoduodenectomy.

Domain	Decision-changing clues and focused assessment	Matched response and escalation	Reassessment priorities and current uncertainties
Muscle loss and functional decline	Decision-changing clues: low grip strength or physical performance; low or declining CT muscle quantity/radiodensity; inactivity; inadequate protein-energy intake; poor treatment tolerance. Focused assessment: review clinically indicated CT consistently and pair it with one repeatable functional test (eg., handgrip, chair-rise, gait speed, 6-min walk). Establish a baseline at diagnosis/start of neoadjuvant therapy and repeat the same functional measure before surgery and during early recovery; record weight/intake trajectory, activity, and symptoms limiting training or eating.	Provide individualized protein-energy support, ideally linked to resistance-oriented prehabilitation or rehabilitation, early mobilization, and symptom control. Add oral nutritional supplements when food intake is insufficient; consider enteral support if a clinically important deficit persists. Escalate when function, weight, or treatment tolerance continues to deteriorate despite initial support.	Track the same functional measure longitudinally and interpret it with CT muscle trajectory when repeat clinical imaging is available. Concordant decline strengthens concern for progressive sarcopenia; discordant change should trigger review of reversible functional limitations. Major evidence gaps include PD-specific thresholds and the optimal intensity and duration of combined nutrition-exercise programs.
PEI and impaired digestion	Decision-changing clues: steatorrhea, bloating, urgency, frequent stools, postprandial discomfort, weight or muscle loss despite apparently adequate intake, or fat-soluble vitamin deficiency; low intake may mask steatorrhea. Focused assessment: stool and meal history; PERT product, dose, timing with meals/snacks, and adherence; operative anatomy and extent of resection; targeted micronutrients; fecal elastase when clinically informative.	Start or optimize PERT with every meal and snack; teach timing with the first bite and dose titration; review snack coverage and adherence. Consider acid suppression only after checking basic PERT use and dose optimization. Correct documented deficiencies and evaluate alternative causes such as delayed gastric emptying, bile acid diarrhea, bacterial overgrowth, or another gastrointestinal disorder. Escalate when symptoms or tissue loss persist despite optimized therapy.	Review stool pattern, meal tolerance, weight or muscle stabilization, micronutrient status, adherence, and downstream glycemic changes. Important uncertainties include the best post-PD diagnostic strategy for PEI and the most appropriate enzyme-dosing algorithms after pancreatic resection.
Inflammatory and catabolic burden	Decision-changing clues: rapid involuntary loss of weight or function, anorexia, elevated CRP in context, cholangitis or other infection, biliary obstruction, treatment toxicity, postoperative complications, or continued muscle loss despite apparently reasonable intake. Focused assessment: determine whether the pattern is acute with an identifiable reversible source or persistent after source control; review serial weight/intake, function, disease stage and treatment phase, CRP in context, pain, nausea, early satiety, delayed gastric emptying, mood, and other reversible barriers.	For an acute reversible stressor, prioritize source control and symptom treatment while limiting further nutritional deficit and maintaining protein-energy delivery as feasible. For persistent cachexia after reversible drivers are addressed, use multimodal supportive care with goals of function, symptom relief, treatment tolerance, and realistic stabilization rather than weight restoration alone. Use enteral or parenteral nutrition when a clinically important deficit is expected or present and the route fits the treatment plan.	Assess resolution of the acute source, intake adequacy, symptom relief, weight and functional trajectory, and ability to proceed with planned therapy. Persistent decline after source control should trigger reassessment for progressive disease, PEI, other symptoms, or persistent or advanced cancer-associated cachexia. Better tools are needed to distinguish reversible catabolism from treatment-resistant cachexia.
Glycemic instability and pancreatic endocrine insufficiency	Decision-changing clues: pre-existing or new-onset diabetes, stress hyperglycemia, marked variability, recurrent hypoglycemia, or changing insulin needs as intake, steroids, PERT, infection status, feeding route, or extent of resection changes. Focused assessment: daily glucose pattern and HbA1c when interpretable; insulin/other therapy; feeding route and timing; steroid exposure; renal function; PEI and PERT use. Consider CGM particularly in insulin-treated patients with recurrent/suspected hypoglycemia, marked variability, rapidly changing insulin needs, or extensive/total resection.	Coordinate nutrition delivery with individualized medication or insulin adjustment; avoid unnecessarily restrictive diets in malnourished patients; revise regimens promptly when absorption or feeding route changes. Even when glycemia is prioritized, concurrently review intake, PEI/PERT, infection/inflammation, renal function, steroids, and feeding route. Seek early endocrine support for recurrent hypoglycemia, persistent hyperglycemia, marked variability, or pancreatogenic diabetes.	Monitor glucose profile or time in range when CGM is used, hypoglycemia burden, achieved intake, changing insulin requirements, and ability to self-manage after discharge. CGM is not required routinely in stable non-insulin-treated patients without substantial variability. Evidence remains limited for PD-specific nutrition-glucose algorithms and optimal CGM deployment after partial resection.

Domains frequently overlap. Prioritization identifies the leading treatment target, responsible clinician, escalation rule, and reassessment date, but it does not suspend assessment of the other domains. At each review, a core cross-domain review of intake/symptoms, function, digestion/PERT, inflammatory context, and glucose should be retained, with immediate parallel management of infection, obstruction, severe glycemic instability, or major feeding failure.

## 3 Four treatment-oriented domains

### 3.1 Muscle loss and functional decline

CT-derived muscle measures are consistently associated with outcome in pancreatic cancer, although thresholds and methods remain heterogeneous (11–14). They are most useful when interpreted with strength or performance rather than as stand-alone labels. Prospective pancreatic-resection data combining CT-defined muscle quantity with handgrip strength, gait speed, or sit-to-stand testing support the complementary value of structural and functional assessment (15). Low muscle area with preserved function may indicate vulnerability without current functional failure, whereas declining strength or performance despite stable CT should prompt evaluation of deconditioning, symptoms, inadequate intake, and treatment toxicity. Frailty, sarcopenia, and cachexia overlap but are not interchangeable (16–18).

For longitudinal monitoring, use the same simple functional test at diagnosis or start of neoadjuvant therapy, before surgery, and during early recovery; handgrip strength or chair-rise testing is often practical, with gait speed or 6-min walk distance used when workflow permits. Repeat CT muscle measures on clinically indicated scans rather than adding imaging solely for nutrition surveillance. Emphasize trajectory and concordance: parallel decline in CT muscle and function strengthens concern for progressive sarcopenia, whereas discordant change should trigger review of pain, neuropathy, fatigue, activity, intake, and other reversible causes. Deterioration during neoadjuvant treatment has been associated with difficulty continuing postoperative chemotherapy (19–22).

The response should combine protein-energy support with a muscle stimulus. Nutrition alone is unlikely to correct functional decline, while exercise is difficult to sustain when nausea, pain, or poor intake remain untreated. Prehabilitation studies in pancreatic and hepatobiliary surgery show that functional improvement is feasible, but no single protocol is clearly superior (23–27). The near-term target is stabilization or recovery of the selected functional measure and treatment tolerance; CT trajectory can provide complementary information when follow-up imaging is already clinically indicated.

### 3.2 PEI and impaired digestion

PEI may precede surgery because ductal obstruction and loss of functioning parenchyma already impair digestion. After PD, altered anatomy and reduced enzyme delivery make it even more common. The reported incidence varies substantially because studies use different tests and definitions, but prospective data confirm that exocrine function often deteriorates after resection (28–30). A digestion-focused assessment is warranted when steatorrhea, bloating, urgency, frequent stools, postprandial discomfort, or continued tissue loss occurs despite apparently adequate intake. Classic steatorrhea may be absent when the patient has already reduced fat or total food intake.

Fecal elastase is a reasonable initial test in an appropriate setting, although its limitations after pancreatic surgery should be recognized. When symptoms are typical and the pretest probability is high, pancreatic enzyme replacement therapy (PERT) can be started while competing causes of diarrhea or maldigestion are considered; improvement with PERT does not establish the diagnosis on its own (31). In practice, treatment failure often reflects technique rather than pharmacology. The product, lipase dose, timing with the first bites of food, use with snacks, and adherence should all be checked before concluding that PERT is ineffective (32–36).

Persistent symptoms should trigger a structured review rather than repeated empiric calorie escalation. Delayed gastric emptying, bile acid diarrhea, small-bowel bacterial overgrowth, and other gastrointestinal disorders may coexist with PEI. Acid suppression can be considered in selected patients whose response remains inadequate despite appropriate timing and dose titration, but it should not replace correction of basic prescribing and administration errors. Follow-up should focus on meal tolerance, stool pattern, weight or muscle trajectory, documented micronutrient deficiencies, and glycemic consequences, since improved absorption may alter insulin requirements (37).

### 3.3 Inflammatory and catabolic burden

The inflammatory and catabolic domain includes rapid involuntary loss of weight or function with infection, biliary obstruction, treatment toxicity, postoperative complications, or progressive cancer. Clinically, an acute, potentially reversible inflammatory stressor should be distinguished from persistent cancer-associated cachexia (38–43). An acute stressor usually has a recent onset, an identifiable source, and a trajectory expected to improve after source control. Persistent weight or muscle loss after reversible stressors have been addressed should prompt assessment for cancer-associated cachexia using longitudinal weight or body-composition change, anorexia and functional decline, and the broader cancer and treatment context; no single CRP threshold establishes this distinction.

Assessment should integrate weight and function with actual intake, gastrointestinal symptoms, disease stage and treatment phase, and reversible contributors such as pain, nausea, delayed gastric emptying, oral problems, and low mood. CRP is useful as contextual evidence rather than a nutrition target. During cholangitis or another acute infection, nutritional goals are to limit further deficit, maintain protein-energy delivery as feasible, and support the patient through source control. When a persistent cachexia pattern remains after reversible drivers are addressed, goals shift toward preserving function, relieving symptoms, supporting treatment tolerance, and achieving realistic stabilization rather than assuming that calorie escalation alone will restore lost tissue (38–40, 44).

Weight loss during neoadjuvant therapy should prompt clinical interpretation rather than automatic prognostic labeling. A loss of at least 5% was common but not independently associated with survival in one cohort (45), whereas baseline nutritional status in advanced PDAC has been associated with chemotherapy delivery (46). Enteral or parenteral nutrition is appropriate when a clinically important deficit is expected or present and the route fits the treatment plan (47, 48). Continued decline after source control and appropriate supportive care should prompt reassessment for progressive disease, PEI, uncontrolled symptoms, or persistent or advanced cancer-associated cachexia instead of repeated empiric calorie escalation.

### 3.4 Glycemic instability and pancreatic endocrine insufficiency

Glycemic instability before and after PD reflects more than the presence or absence of diabetes. Pancreatic reserve, steroid exposure, infection, feeding route, nutrient absorption, and insulin requirements can change rapidly. Relevant patterns include stress hyperglycemia, recurrent hypoglycemia, marked variability, and new or changing insulin needs after resection. Extent of resection is important: when a planned PD is extended or converted to total pancreatectomy, complete endocrine deficiency creates a substantially greater insulin-management and hypoglycemia burden than after standard PD (49–51). Perioperative evidence also cautions against indiscriminate near-normal glucose targets because intensive control increases hypoglycemia risk (52).

CGM need not be deployed routinely in every patient undergoing PD; however, it should be considered or offered when insulin therapy is required, particularly in patients with recurrent or suspected nocturnal/asymptomatic hypoglycemia, marked glycemic variability, rapidly changing insulin requirements during feeding transitions, or extensive/total pancreatic resection. Studies after total pancreatectomy show a substantial hypoglycemia burden and demonstrate that CGM can quantify time in range, variability, and previously unrecognized low glucose, although evidence for improved clinical outcomes remains limited (50, 51). Current diabetes standards support CGM for adults treated with insulin (53). Stable patients not receiving insulin and without substantial glycemic variability can usually be followed with conventional glucose monitoring.

Even when glycemia is the immediate priority, it should never be treated as an isolated domain. Each review should concurrently check actual carbohydrate and protein-energy delivery, PEI symptoms and PERT use, infection or inflammatory activity, renal function, corticosteroids, and feeding route because changes in absorption or catabolism can alter insulin needs within days. Restricting intake simply to suppress hyperglycemia may deepen a nutritional deficit, whereas failure to reduce insulin after interrupted feeding or altered absorption can precipitate hypoglycemia. Recurrent hypoglycemia, persistent hyperglycemia, or marked variability warrants early endocrine input and prompt reassessment of the interacting nutrition domains.

## 4 Applying the framework across treatment stages

At diagnosis and during neoadjuvant therapy, a positive nutrition screen should be followed by a compact domain-oriented assessment: recent weight and intake, gastrointestinal symptoms and PERT use, CT muscle review when available, one functional test, glucose status, and inflammatory context when clinically relevant. Clinical stage and treatment intent should be recorded because the time available for optimization differs between upfront surgery and neoadjuvant treatment. The next oncologic milestone determines the leading priority, but the core cross-domain assessment is repeated so that a new problem is not missed when attention is focused elsewhere.

This approach does not depend on sophisticated technology. The core assessment can be collected in routine surgical and oncology clinics. Specialized testing is reserved for unresolved diagnostic uncertainty or failure to respond. What matters is whether the assessment changes care. Each prioritized domain should have a designated clinician or team, an escalation rule, and a reassessment date. Without those elements, repeated screening risks documenting deterioration without changing management.

During the preoperative interval and postoperative admission, domain-guided care is most useful when routine pathways are no longer sufficient. Early oral feeding remains appropriate for many patients, but it will not meet the needs of every nutritionally vulnerable patient after PD. Comparative studies and a recent Cochrane review support selective rather than universal escalation of postoperative nutrition support (54–58). The NUTRIWHI randomized trial suggests that early supplemental nasojejunal feeding may reduce complication burden in nutritionally at-risk patients, but it does not justify routine tube feeding for all patients undergoing PD (55). Evidence for immunonutrition remains heterogeneous (59, 60). Enhanced recovery pathways improve recovery overall, yet they do not remove the need for individualized escalation when intake, digestion, function, or glycemic control deteriorates (61, 62).

After discharge, we favor structured review at approximately 2 and 6 weeks, with earlier contact for major symptoms, feeding failure, infection, or severe glycemic instability. These time points are pragmatic rather than validated. They coincide with a period in which cumulative deficits, unrecognized PEI, and delayed recovery often become apparent, and when preparation for adjuvant therapy is taking shape. The review should ask whether the patient can eat, digest, mobilize, and manage glucose sufficiently well to continue the cancer pathway. “Readiness” is not a single laboratory value; it means that unresolved complications, uncontrolled symptoms, major nutritional deficits, or unstable glycemia are not silently delaying the transition to oncology care.

Responsibility becomes particularly important at transitions of care. PEI, glucose instability, and progressive weakness frequently emerge or worsen after discharge, when ownership may be divided among surgical, oncology, nutrition, and primary care teams. A domain should not be considered addressed simply because it appears in the discharge summary. The handover needs to specify who will adjust PERT, who will review glucose therapy, what change should trigger earlier contact, and when function and intake will be reassessed.

## 5 Research priorities

The framework now requires prospective testing. A pragmatic multicenter trial could compare standardized perioperative nutrition care with a pathway in which screening is followed by domain-oriented assessment, assignment of one or more priority targets, and a documented reassessment plan. The relevant comparison is not nutrition versus no nutrition. It is generic nutritional support versus support linked explicitly to the leading mechanism of decline. Trial fidelity should be assessed by recording what patients actually receive: energy and protein delivery, exercise exposure, PERT dose and timing, symptom treatment, feeding route, and glycemic exposure.

Outcomes should be measured at three levels. Domain-specific responses include preservation of strength, improved meal tolerance after PERT, resolution of a reversible inflammatory source, or reduced glucose variability after medication is matched to intake. Recovery outcomes include complication burden, unplanned healthcare use, and patient-reported symptoms. Oncologic outcomes include time to adjuvant therapy and completion of planned chemotherapy. Digital food records, automated CT segmentation, and wearable activity monitoring may support implementation, but the resulting algorithms must remain interpretable and subordinate to clinical judgment.

## 6 Discussion

The framework does not introduce new measurements. Its purpose is to organize established assessments around explicit, revisable treatment priorities while maintaining surveillance of coexisting problems. This may help address two practical failures: stopping at a risk label without identifying a treatable mechanism, and allowing the most visible problem to obscure another changing domain. Important limitations remain: the domains overlap, specialist access is uneven, and several thresholds and reassessment intervals have not been prospectively validated. The framework should therefore be evaluated as a clinical decision scaffold rather than adopted as a new diagnostic classification.

For patients with PDAC undergoing PD, nutrition care is unlikely to change tumor biology, but it can reduce avoidable deficits, improve symptom control, preserve function, and support delivery of intended oncologic treatment. Risk scores remain useful for identifying who needs attention. They should not be the endpoint of assessment. The clinically consequential question is what is driving decline in this patient, at this stage, and what response should be visible before the next treatment milestone.

## Data Availability

The original contributions presented in the study are included in the article/supplementary material, further inquiries can be directed to the corresponding authors.
